# Saliva Metabolomic Profile in Dental Medicine Research: A Narrative Review

**DOI:** 10.3390/metabo13030379

**Published:** 2023-03-03

**Authors:** Konstantinos Tzimas, Eftychia Pappa

**Affiliations:** Department of Operative Dentistry, School of Dentistry, National and Kapodistrian University of Athens, 11527 Athens, Greece

**Keywords:** metabolomics, saliva, biofluid, metabolome, biomarker, oral cancer, periodontitis

## Abstract

Metabolomic research tends to increase in popularity over the years, leading to the identification of new biomarkers related to specific health disorders. Saliva is one of the most newly introduced and systematically developed biofluids in the human body that can serve as an informative substance in the metabolomic profiling armamentarium. This review aims to analyze the current knowledge regarding the human salivary metabolome, its alterations due to physiological, environmental and external factors, as well as the limitations and drawbacks presented in the most recent research conducted, focusing on pre—analytical and analytical workflows. Furthermore, the use of the saliva metabolomic profile as a promising biomarker for several oral pathologies, such as oral cancer and periodontitis will be investigated.

## 1. Introduction

Saliva is the readily accessible biological fluid of the oral cavity [1]. It is produced by three major pairs of glands and hundreds of minor ones. The three major glands participating in the production of saliva are the parotid, submandibular and sublingual glands, which lead to the classification of parotid saliva, submandibular saliva and sublingual saliva respectively [2]. Additional classification of the secretion of saliva is based on the presence or absence of stimuli leading to its production. More precisely, at rest, *unstimulated saliva* is produced with its major portion originating from the submandibular glands (almost 70%), whereas *stimulated saliva*, induced by stimuli such as smell, taste ordrugs originates primarily from the parotid glands [1,3].

The term “whole mouth saliva” (WMS) is used in oral sciences to determine the transparent, clear, watery fluid composed by a mixture of the parotid saliva, the submandibular saliva and the sublingual saliva, combined with the secretions of minor salivary glands, the gingival crevicular fluid, eukaryotic cells (epithelial as well as leukocytic), food debris, microorganisms and their metabolites [4,5,6].

Due to its aqueous composition, saliva mainly consists of water (99% of its composition). Further components, such as mucus, digestive enzymes, growth factors, cytokines, immunoglobulins, antibacterial peptides, salts and low molecular weight metabolites are numbered among salivary products [7].

**Figure 1:**
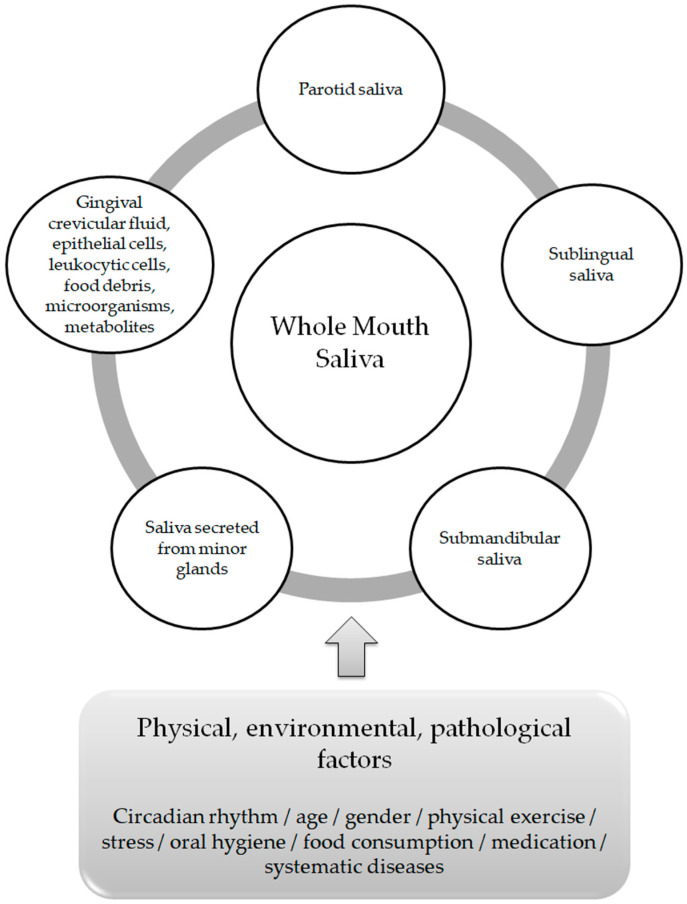
Whole Mouth Saliva components and factors affecting its composition.

The average healthy person generates 0.75 to 1.5 L of saliva per day, with a greater volume secreted when the person is awake [8]. There is an evident differentiation in the composition, flow rate and volume of saliva both between individuals and within the same individual [9]. These variations depend on stimuli delivered by the sympathetic and parasympathetic systems of the autonomic nervous system (neural control) [10], as well as on physical, environmental and/or pathological factors, which include circadian rhythm, age, gender, physical exercise, oral hygiene, food consumption, medication andsystematic diseases [11] (Figure 1). Among its functions, lubrication and moisturization of the surfaces of the oral cavity, the pharynx and the esophagus, oral digestion, tissue and tooth integrity protection and antibacterial and antiviral defense, play a pivotal role in oral homeostasis and in the overall quality of life [12,13].

Biotechnological advances and applications in the health sciences have led to the introduction of “omics” in medicine. Genomics, studying the structure, function, evolution and mapping of genes and transcriptomics, the field of biological study of mRNA molecules led to the formation of the appropriate conditions for circumstantial monitoring of smaller organ and/or cell compounds such as proteins (proteomics) and low molecular weight metabolites (metabolomics) [14,15].

A metabolite is a small molecule with a molecular weight typically less than 1500 Da [16]. The complete set of small molecular metabolites is called the “metabolome” [17]. “Metabolomics” is defined as the latest of the –omics technologies, investigating metabolites within biofluids, cells and tissues [18].

Τhe use of biofluids in the human body such as serum, plasma, urine and cerebrospinal fluid for metabolomic profiling on a variety of health disorders including cancer, infectious diseases, neurological diseases (Alzheimer’s disease, dementia) cardiovascular, rheumatological, renal, and respiratory diseases is scientifically established and acceptable [19]. On the contrary, scarce evidence is present when addressing salivary metabolites [20]. The salivary metabolome is considered as a critical asset in elucidating pathways identifying various local and systematic disorders, and it may be used as a key mediator in treatment design and modification as well as in treatment outcomes [21].

The aim of this narrative review is firstly, to shed light on the importance of the human salivary metabolome in health, secondarily to assess the analytical protocols and the limitations of salivary metabolomic studies and lastly to analyze the salivary metabolomic profile as a possible, sufficient and powerful biomarker of oral pathogenesis.

## 2. Human Salivary Metabolome Research and Its Limitations

A lot of studies focus on metabolite profiling in blood and urine, whereas scientific evidence for metabolic profiling in saliva is lacking. Takeda et al., concluded that the saliva metabolome proved to be comparable to the human serum and cerebrospinal fluid metabolomes in terms of chemical composition, strengthening the belief of homogeneity of compounds found in human saliva and human blood, independently of their different concentrations [20]. The well known positive correlation between salivary and plasma metabolite levels (e.g., glucose, lactate and pyruvate), as well as the fact that the proteomic and metabolomic alterations observed in saliva follow a similar pattern to the changes seen in blood, reinforce the use of saliva as an informative diagnostic biofluid [22,23].

Among the benefits of saliva are its ad libitum production, non-invasiveness, painlessness, relatively fast and cheap collection, minimal collector training, reduced anxiety when compared to blood collection and child friendlier approach when compared to blood collection, making it the perfect, informative, most readily available biofluid [5,24,25,26] The convenient analysis of saliva samples, the non-infectious collection process, the ease of its transportation and its disposable nature are further positive aspects of using saliva for metabolite profiling processes [27].

Most salivary metabolomic research on healthy subjects focuses on the identification of specific metabolites or metabolic species. This kind of research is characterized as *targeted salivary metabolomic research* [23]. The first important *non—targeted metabolomic analysis of saliva* was conducted by Silwood et al., in 2002, who identified more than 60 metabolites and quantified 11 salivary metabolites in healthy human saliva, along with an interesting intra and inter—subject variability in the concentrations of these molecules [28]. Likewise, Sugimoto et al. (2013) identified and quantified 148 salivary metabolites in healthy humans [29]. Dame et al. (2015) accomplished the identification and the quantification of a total of 308 salivary metabolites in healthy people [23]. Nowadays, more than 853 identified and quantified salivary metabolites or metabolite species are freely available at the Human Metabolome Database (HMDB) [30].

All salivary metabolomic research studies (either targeted or non-targeted) of healthy human samples, focus on multiple factors that tend to modify the concentration of the healthy saliva metabolome. The greatest factors affecting the healthy human saliva metabolome are: *the collection method*, where stimulated saliva presents a decrease in metabolite concentrations compared to unstimulated whole mouth saliva secretion samples [20]; *the type of the gland that the saliva is secreted from,* since submandibular gland saliva is more viscous than the serous parotid gland saliva [30]; *the gender*, where acetate, formate, glycine, lactate, methanol, propionate, propylene glycol, pyruvate and taurine were significantly higher in concentration in male rather than in female saliva samples [20,31], *the smoking status*, that leads to up- and/or down-regulations of metabolic concentrations [32], *the diurnal cycle* (circadian cycle), where specific salivary metabolites—mainly amino-acids- showed a clear diurnal variation in their concentration [33], *the fasting conditions (diet)*,where longer time period between last diet and sample collection affected the salivary metabolomic profile [23,34] and the *microflora* of the oral cavity, but most precisely the *host-microbiome interactions*. The oral microbiome strongly affects the net metabolic composition of the WMS at rest and can lead to alterations in its composition upon exposure to exogenous substances. The updated literature indicates that certain WMS metabolites including short chain fatty Acids (SCFAs), are absent from the sterile parotid gland saliva, leading to the conclusion that some saliva metabolites present a strong correlation with the bacterial index of the WMS. Furthermore, the metabolic patterns of the WMS present greater inter—individual variations than those of plasma metabolites, possibly caused by the existence of diversity in the oral microbiota that modulates the WMS metabolites. Conversely, plasma metabolites are easily regulated due to host mechanisms [26,35,36]. A field of further investigation involves the reflection of the oral microbiome on the salivary metabolome, as well as the dynamic interactions of different biofluids.

**Figure 2:**
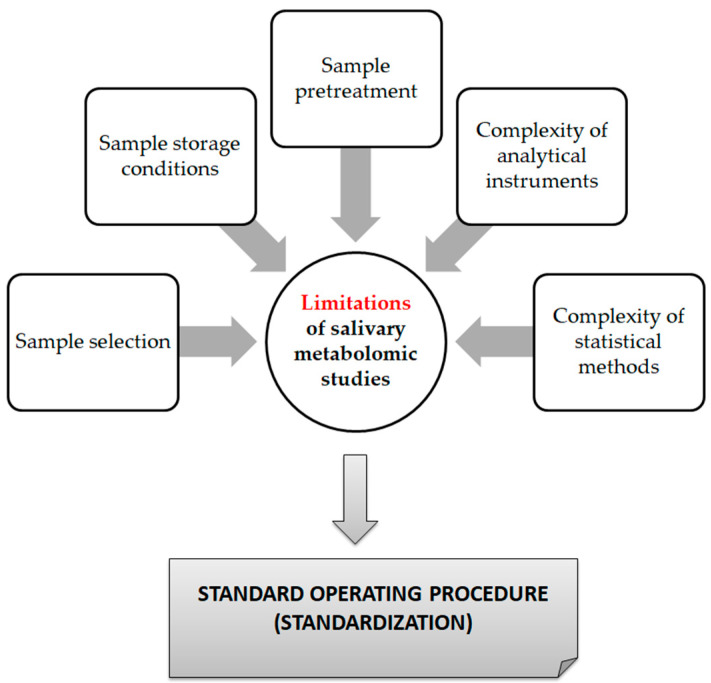
Limitations of saliva metabolomic profile studies.

**Figure 3:**
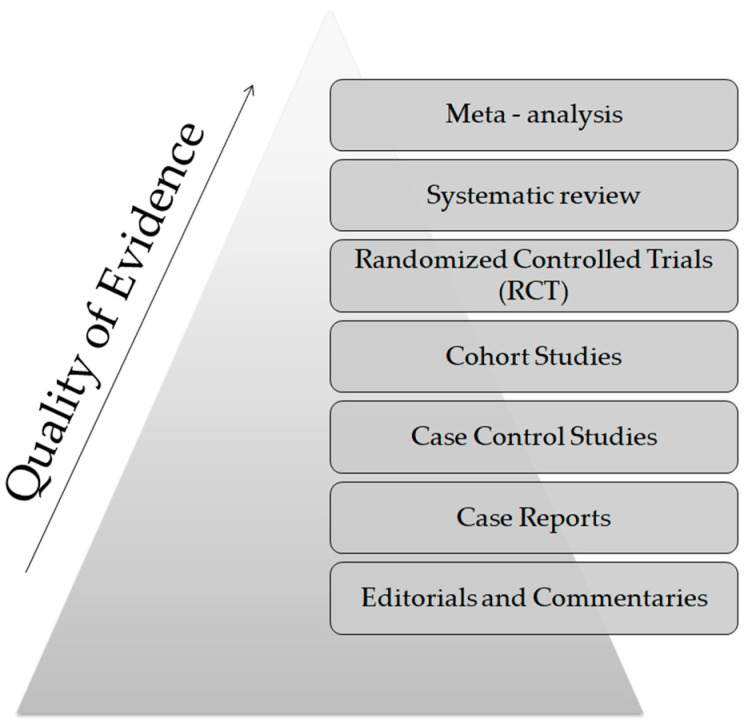
The evidence—based pyramid.

From a technical point of view, several analytical platforms are developed and integrated into the human saliva metabolomic profiling process. The two most renowned metabolite measurement technologies are nuclear magnetic resonance spectroscopy (HNMR) and mass spectrometry (MS) [20,23,24,37,38]. Subcategories of MS methods or additional combinatorial/conjunctive methods are mentioned below. Capillary electrophoresis time-of-flight mass spectrometry (CE-TOF-MS), gas-chromatography mass spectrometry (GC-MS), direct flow injection—liquid chromatography mass spectrometry (LC-MS), inductively coupled plasma mass spectrometry (ICP-MS) and high performance liquid chromatography (HPLC) [23,39,40]. Τhe description of each analytical platform is outside the scope of this mini-review. NMR is characterized as an untargeted metabolomics technique that leads to the identification and quantification of compounds, including short-chain organic acids, amino acids, alcohols, amines, sugars and pharmaceutical adjuvants. The advantage of this technique focuses on the minimal or no sample pre-treatment needed (deproteinization by centrifuging) and on its higher reproducibility compared to MS analytical platforms [41]. On the other hand, MS is an analytical method of high sensitivity, that identifies and/or quantifies a substance by measuring its mass and number of ions by the use of various ionization methods [42]. The combination of MS with other conjunctive methods has the advantage of greater metabolite identification even at lower concentrations [43,44,45]. The use of complex extraction methods and separation steps in order to detect and analyze both polar and non-polar organic acids is highlighted as a difficulty that complicates the identification procedure by mass spectrometry [41]. MS-based metabolomics require a well-designed pooled quality control sample (PQC) that is repeatedly analyzed throughout the sample batch and used for signal corrections compared to NMR-based metabolomic analysis [18,46]. Those characteristics are mentioned in order to understand that an additional limitation in salivary metabolomic studies exists due to the complexity of the analytical instruments used. Most studies only use one analytical platform and try to analyze one individual metabolome, which makes a potential comparison of salivary metabolic profiles between studies using different analytical technologies insignificant. At this point the allusion to the term “*standard operating procedure*” is of utmost importance (Figure 2). This term refers to the standardization and enactment of specific workflows as to the pre-analytical, analytical and post-analytical methods used [47,48]. The standardization of sample collection (for example, the use of unstimulated WMS in all studies), sample storage conditions (freezing temperatures), sample pretreatment (centrifugation for cell content removal and/or additional separation steps), sample analysis (by the use of more than one analytical platform) and statistical methods employed (principal component analysis—partial least-squares regression) would minimize the heterogeneous results in salivary metabolomic coverage [13,25,26], caused by separation difficulties, sensitivity differences, instrument detection differences, compound stability, solubility, and volatility [23,49,50], and give the opportunity to conduct comprehensive systematic reviews and meta-analyses, which are placed on top of the evidence-based science pyramid (Figure 3). Scientific studies are categorized based on the quality as well as the amount of evidence available, meaning that towards the base of the pyramid, the amount of evidence increases but simultaneously the quality of the evidence decreases.

## 3. The Human Salivary Metabolome and Oral Pathologies

According to the World Health Organization (WHO), a “biomarker” is defined as any substance, structure, or process that can be measured in the body or in its products and that can influence or predict the incidence of an outcome or disease [51]. Over the years, clinical studies have focused on detecting specific salivary metabolites associated with specific oral diseases, thus characterizing these metabolites as diagnostic biomarkers. This remains questionable, as one salivary metabolite’s quantitative alteration may simply indicate a non-specific pathological shift, incidental to insufficient diagnostic specificity. On the other hand, multivariate analysis may offer greater accuracy for putative biomarker findings [26]. The early “fingerprints” of changes in a wide range of diseases could be discovered by the study of the metabolic profile of saliva. Focusing on oral pathology and its association with salivary metabolomics, efforts have been made in the fields of oral cancer and periodontal diseases.

As mentioned by Khurshid et al., in 2018, oral cancer is the 6th most common cancer worldwide, and its late detection is highly associated with its high mortality and morbidity rates. Approximately 60–80% of patients with oral cancer are diagnosed at a late-stage. Hundreds of salivary biomarkers (using genomics, transcriptomics and proteomics) have already been identified, including cytokines (IL-8, IL-1b, TNF-α), defensin-1, P53, tissue polypeptide-specific antigen, dual specificity phosphatase, profilin, cofilin-1, transferrin etc. [52]. Human saliva metabolomics and its contribution to oral cancer diagnosis is a field of continuous investigation, since saliva is in direct contact with the mucosa and cancerous cells of the oral cavity. The validation of specific saliva metabolomic biomarkers for oral cancer may lead to early stage detection and a more appropriate treatment modality for the patient [13,21,25,26]. Several studies, with different protocols, conducted over the last few years identified different saliva metabolite concentrations either between healthy controls and oral squamous cell carcinoma subjects or between OSCC subjects and premalignant lesion subjects (oral lichen planus, oral leukoplakia, precancerous dysplasia, keratosis). Their samples, analytical and discrimination methods and outcomes are summarized in Table 1 [53,54,55,56,57,58,59,60,61,62,63,64,65,66,67]. A notable diversity of candidate biomarkers is presented in these studies. More precisely, Sugimoto et al. (2010) [55] and Wang et al. (2014) [57,58] both mentioned increased choline and betaine and decreased L-carnitine in patients with OSCC compared to healthy controls. Choline is also found among other metabolites as a potential biomarker in the research of Ohshima et al., in 2017 [60]. Glycine, proline and ornithine were found in three independent studies by Lohavanichbutr et al. (2018), Ishakawa et al. (2019) and Tantray et al. (2022) as potential oral cancer biomarkers [61,62,67]. However, different studies identified different groups of metabolites that were either upregulated or downregulated in OSCC and precancerous samples compared to healthy controls. These deviations are associated with limitations concerning sample size, population tested, and analytical methods used—factors that lead to heterogeneous results despite the similar study designs of the studies. The limitations of this kind of study lie in two factors: firstly, the evaluation of the specificity of the salivary metabolite biomarker against other inflammatory diseases, for instance, periodontitis, because a possible overlap in the metabolite biomarkers between periodontitis and oral cancer could lead to serious misdiagnosis, and secondarily, the study design, the analytical instruments used, the discrimination methods, and the absence of cross-validation of the analytical equipment between different laboratories [13].

The same opportunities, but simultaneously the same concerns, are detected in the use of salivary metabolites in periodontitis diagnosis and treatment. Periodontal diseases are characterized as one of the two main causes of tooth loss and are inextricably linked to connective tissue loss, periodontal pocket formation, and progressive bone degradation [21,68]. The development of new saliva metabolite biomarkers may eliminate tooth loss due to early diagnosis of the severity of the periodontal condition. In several studies that are listed in Table 2, an upregulation or downregulation of specific salivary metabolite species is detected (e.g., increased levels of fatty acids, phenylphenol, dipeptides leucylisoleucine, serylisoleucine, arachidonate, and dihomo-linolate) [55,69,70,71,72,73,74,75,76,77,78,79,80,81,82,83]. Besides saliva, the gingival crevicular fluid (GCF) is a great source of possible biomarkers for periodontal diseases. It includes a variety of host and microbial enzymes, endotoxins, nucleic acids, carbohydrates, lipids, degradation products of several metabolic pathways, cytokines and immunoglobulins. It is revealed that GCF is a more ideal biofluid for the diagnosis of periodontal diseases (and the differentiation of healthy patients, gingivitis patients, and periodontitis subjects) compared to serum and saliva, since it contains biomarkers that reflect inflammation, immune response and tissue destruction at the sight of periodontal lesions [84]. Metabolites associated with periodontal variables are clearly linked to tissue destruction, host defense mechanisms and bacterial metabolism [76]. When interpreting the presence of “salivary metabolites” in periodontitis patients, caution is needed, because they may be products of the developed microflora (oral microbiome metabolites). The bacterial metabolite phenylacetate is strongly correlated with periodontal disease. Salivary metabolomics may also be used as prognostic biomarkers of non-surgically and/or surgically treated periodontal disease. A change in the salivary metabolomic profile of a diseased patient after treatment entails the modification of a previously “diseased” specimen into a newly “healthy” one. 

## 4. Conclusions

Conclusively, salivary metabolomics form a promising newly established research field. The drawbacks of using saliva metabolites as putative biological indicators of oral or systematic health disorders focus on the small sample sizes of the studies conducted and on the great challenges of the implementation of these technologies into clinical diagnostics. The validation of salivary biomarkers may be accomplished by elevating metabolomic research from a “case-control-study” design to a “large-scale validation study” design. The simultaneous observation of salivary metabolomics and microbiomics would enlighten the pathogenetic mechanisms of oral diseases. The primary goal of salivary metabolomic profiling is to distinguish the type of inflammation itself and not to simply compare an inflammation status to that of a healthy control. Salivary metabolomics may open new horizons in clarifying pathogenesis, as well as in disease monitoring and treatment outcome assessment. Mapping the human saliva metabolome of individuals at many standpoints is equivalent to the establishment of more personalized treatment and follow-up protocols. All in all, the oral cavity is a complex organ with numerous factors affecting its salivary metabolic profile, a fact that may ramify accurate research findings.

## Figures and Tables

**Table 1:** Research focused on salivary metabolomics serving as biomarkers in oral cancer.

Authors	Groups	Analytical Method	Discrimination Method	Observations—Candidate Biomarkers
Yan et al., 2008 [53]	Oral squamous cell carcinoma (OSCC)/precancerous lesions	High performance liquid chromatography—mass spectrometry (HPLC—MS)	Multivariate hierarchical principle component analysis	
Jou et al., 2010 [54]	Healthy controls vs. oral cancer subjects	Matrix-assisted laser desorption/ionization time-of-flight mass spectrometry (TOF–MS)	Western plotting + immunoassays	Increased transferrin levels in oral cancer subjects + linear correlation between transferrin and stage of tumor
Sugimoto et al., 2010 [55]	Healthy controls vs. oral cancer vs. pancreatic cancer vs. breast cancer vs. periodontal disease	Capillary electrophoresis time-of-flight mass spectrometry (CE-TOF-MS)	Principal component analysis (PCA)/independent multiple logistic regression models (MLR)	Increased taurine, choline, and betaine Decreased pipecolinic acid and L-carnitine
Wei et al., 2011 [56]	Healthy controls vs. oral cancer vs. precancerous conditions	Ultraperformance liquid chromatography coupled with quadrupole time-of-flight mass spectrometry (LC—TOF—MS)	PCA/orthogonal partial least squares discriminant analysis	Increased valine, lactic acid and phenylalanine are the best combination of salivary biomarkers for the discrimination of oral cancer from precancerous conditions
Wang et al., 2014 [57]	Healthy controls vs. Stages I + II OSCC	LC—MS analysis	Multivariate data analysis	Increased choline, betaine and pipecolinic acid Decreased L—cainitine in OSCC
Wang et al., 2014 [58]	Follow-up study	Reverse-phase liquid and hydrophilic interaction chromatography and TOF—MS	Multivariate data analysis	Increased propionylcholine decreased N-actyl-L-phenylalanine, sphinganine, phytosphingosine and S-carboxymethyl-L-cysteine
Ishikawa et al., 2016 [59]	Healthy controls vs OSCC	CE—TOF—MS	MLR/support vector machine-feature selection/stepwise feature selection	Increased pipecolate and s-adenosylmethionine
Ohshima et al., 2017 [60]	Healthy controls vs. OSCC	CE—TOF—MS	Wilcoxon rank sum test	Increased choline, valine, isoleucine, leucine, 2-oxoisovaleric acid and 3-hydroxybutyric acid Decreased urea
Lohavanichbutr et al., 2018 [61]	Healthy controls vs. OSCC vs. oropharyngeal squamous cell carcinoma (OPSC)	Nuclear magnetic resonance spectroscopy (NMR), LC—MS, Quadrupole time-of-flight liquid chromatography—mass spectrometry (Q—TOF—LC—MS)	MLR	Decreased glycine, proline, citrulline, and ornithine were associated with early stage OSCC
Ishakawa et al., 2019 [62]	OSCC vs. oral epithelial dysplasia (OED) vs. persistent suspicious oral mucosal lesions (PSOML)	CE—TOF—MS	MLR	Decreased ornithine, carnitine, arginine, o-hydroxybenzoate, N-acetylglucosamine-1-phosphate, and ribose 5-phosphate (R5P) in OSCC/OED compared to PSOML
Shridharan et al., 2019 [63]	Healthy controls vs. oral leukoplakia vs. OSCC	Quadrupole time-of-flight liquid chromatography—mass spectrometry (Q—TOF—LC—MS)	ANOVA/chi—square tests	Increased 1-methylhistidine, inositol 1,3,4-triphosphate, d-glycerate-2-phosphate, 4-nitroquinoline-1-oxide, 2-oxoarginine, norcocaine nitroxide, sphinganine-1-phosphate, and pseudouridine in oral leukoplakia and OSCC decreased l-homocysteic acid, ubiquinone, neuraminic acid, and estradiol valerate.
Ishakawa et al., 2020 [64]	Healthy controls vs. oral cancer	18F-FDG PET/CT	MLR	N-acetylneuraminate and 3-phenylpropionate can be used to discriminate between patients with oral cancer and controls
Song et al., 2020 [65]	Healthy controls vs. OSCC vs. premalignant lesions	Conductive polymer spray mass spectrometry (CPSI-MS)	Lasso regression model	Increased cadaverine, putrescine, spermidine, 5-aminopentanoic acid and proline in the OSCC group Decreased pipecolic acid, lysine, arginine, ornithine, and histidine in the OSCC group
De Sa Alves et al., 2021 [66]	Healthy controls vs. OSCC	Gas-chromatography mass spectrometry (GC-MS)	PCA, Wilcoxon—Mann Whitney test	Identification of 24 metabolites as candidate biomarkers. Increased malic acid, methionine, maltose, and inosine
Tantray et al., 2022 [67]	Healthy controls vs. oral leukoplakia vs. OSCC	GC—MS		Increased decanedioic acid, 2-methyloctacosane, eicosane, octane, 3,5-dimethyl, pentadecane, hentriacontane, 5,5-diethylpentadecane, nonadecane, oxalic acid, 6-phenylundecanea, l-proline, 2-furancarboxamide, 2-isopropyl-5-methyl-1-heptanol, pentanoic acid, docosane

**Table 2:** Research focused on salivary metabolomics serving as biomarkers in periodontal disease.

Authors	Groups	Analytical Method	Discrimination Method	Observations—Candidate Biomarkers
Sugimoto et al., 2010 [55]	Healthy controls vs. oral cancer vs. periodontal disease vs. pancreatic cancer vs. breast cancer	CE-TOF-MS	MLR/PCA	There was no significant differences between patients with periodontal disease and healthy controls concerning oral polyamine levels.
Barnes et al., 2011 [69]	Healthy controls vs. periodontal specimens	UHPLC MS/MS + GC/MS	Welch’s—*t*-test + false discovery rates	Increased dipeptides leucylisoleucine, phenylphenol, serylisoleucine, fatty acids, arachidonate, arachidate Many of these metabolites are the products of host-microbial metabolism.
Aimetti et al., 2012 [70]	Healthy controls vs. gingivitis vs. localized chronic periodontitis vs. generalized chronic periodontitis vs. localized aggressive periodontitis vs. generalized aggressive periodontitis	NMR	PCA, Projection to Latent Structure (PLS), Canonical Correlation Analysis (CA)	Metabolic profiles of generalized chronic periodontitis patients exhibited increased acetate, c-aminobutyrate, n-butyrate, succinate, trimethylamine, propionate, phenylalanine, and valine and decreased concentrations of pyruvate and N-acetyl groups in generalized chronic periodontitis
Huang et al., 2014 [71]	Patients with chronic periodontitis	Inductively coupled plasma mass spectrometry (ICP-MS)/GC-MS/LC-MS	Analysis of variance followed by Student’s *t*-test.	Increased PGE2, PGD2,PGF2a, TXB2, 5-HETE, F2- isoprostane decreased PGI2,13-HODE, and 9-HODE
Barnes et al., 2014 [72]	Diabetic and non-diabetic human subjects with a healthy periodontium, gingivitis and periodontitis	GC-MS/LC-MS	ANOVA/*t*-tests/False discovery rate method	Comparison of healthy, gingivitis and periodontitis saliva samples within the non-diabetic group: Increased levels of oxidized glutathione and cysteine-glutathione disulfide, increased markers of oxidative stress, including increased purine degradation metabolites increased amino acid levels and increased ω-3 (docosapentaenoate) and ω-6 fatty acids (linoleate and arachidonate)
Kuboniwa et al., 2016 [73]	Periodontal inflamed surface area (PISA) before and after removal of supragingival plaque	GC-MS	OPLS	Increased cadaverine, 5-oxoproline, and histidine
Ozeki et al., 2016 [74]	GCF of moderate pockets vs. deep pockets vs. healthy controls	GC-MS	PCA	Increased putrescine, lysine, phenylalanine, ribose, taurine, 5-aminovaleric acid, and galactose in deep pocket sites
Rzeznick et al., 2017 [75]	Healthy controls vs. generalized periodontitis	NMR	PCA/OPLS	Increased short chain fatty acids such as butyrate. Decreased lactate, γ-amino-butyrate, methanol, and threonine
Liebsch et al., 2019 [76]	Age-stratified groups of oral health—correlation between metabolites and periodontal disease severity	LC-MS/MS	Linear regression analysis	Increased phenylacetate.
Singh et al., 2019 [77]	Surgically treated periodontal subjects vs. untreated periodontal patients	NMR	Multivariate and quantitative analysis	Increased lactate, ethanol, succinate, and glutamate in surgically treated periodontal subjects
Romano et al., 2019 [78]	Healthy controls vs. treated generalized chronic periodontitis	NMR	Univariate and multivariate paired approaches	The post-treatment metabolic profile of GCP patients could not be assimilated to that of healthy controls who exhibited different levels of lactate, pyruvate, valine, proline, tyrosine, and formate.
Gawron et al., 2019 [79]	Healthy control vs. chronic periodontitis	NMR	Multivariate analysis/OPLS	Increased lactate and isopropanol decreased glycerol, acetone and methanol
Schulte et al., 2020 [80]	Perinatally-acquired HIV patients vs. HIV-exposed, but uninfected patients and moderate periodontitis	LC-MS/MS		Increased cadaverine particularly in HIV exposed but uninfected individuals with moderate periodontitis
Citterio et al., 2020 [81]	Healthy controls vs. untreated periodontitis vs. non surgically treated periodontal patients	NMR	Multivariate analysis/partial least squares (PLS)/OPLS	The post-NST metabolic profile of periodontal patients could not be completely assimilated to that of healthy controls. decreased leucine, valine, phenylalanine, isoleucine, hypoxanthine and uracil after non surgical treatment compared to untreated periodontitis
Rodriques et al., 2021 [82]	Healthy controls vs. periodontal patients (over 65 years old)	GC-MS	Partial least squares analysis (PLS)	Increased 5-aminovaleric acid and serine in the gingival crevicular fluid
Overmyer et al., 2021 [83]	Supragingival dental plaque of healthy controls vs. periodontitis, vs. periodontitis + diabetes type 2 vs. periodontitis + prediabetes	GC-MS/LC-MS/MS	Generalized additive mixed-effect models/zero-adjusted Gamma distribution, log normal distribution, bimodal log normal distribution/log-likelihood ratio testing/Benjamini–Hochberg FDR correction	Increased phosphatidylcholines, plasmenyl phosphatidylcholines, ceramides containing non-OH fatty acids, and host proteins related to actin filament rearrangement
